# Global asymptotic behaviour of positive solutions to a non-autonomous Nicholson's blowflies model with delays

**DOI:** 10.1080/17513758.2014.917725

**Published:** 2014-05-21

**Authors:** Le Van Hien

**Affiliations:** ^a^ Department of Mathematics, Hanoi National University of Education, 136 Xuan Thuy Road, Hanoi, Vietnam

**Keywords:** Nicholson's blowflies model, positive solutions, exponential convergence, 34C27, 34K14

## Abstract

This paper addresses the global existence and global asymptotic behaviour of positive solutions to a non-autonomous Nicholson's blowflies model with delays. By using a novel approach, sufficient conditions are derived for the existence and global exponential convergence of positive solutions of the model without any restriction on uniform positiveness of the per capita dead rate. Numerical examples are provided to illustrate the effectiveness of the obtained results.

## 1. Introduction

Nicholson [14] used the following delay differential equation:

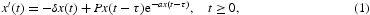

where *a*, δ, *P* and τ are positive constants, to model laboratory population of the Australian sheep-blowfly. Biologically, *x*(*t*) is the size of the population at time *t, P* is the maximum per capita daily egg production rate, 1/*a* is the size at which the population reproduces at its maximum rate, δ is the per capita daily adult mortality rate and τ is the generation time, or the time taken from birth to maturity. The dynamics of Equation (1) was later studied in [5,15], where this model was referred to as the Nicholson's blowflies equation.

The theory of the Nicholson's blowflies equation has made a remarkable progress in the past 40 years and attracted extensive attention from researchers (see, for example, [1] and the references therein). Many important results on the qualitative properties of the model such as existence of positive solutions, positive periodic/almost periodic solutions, persistence, permanence, oscillation and stability for the classical Nicholson's model and its generalizations (in particular, to variable coefficients, time-varying delays and impulsive equations) have been established in the literature [2–4,7–13,16–22].

However, it should be noted that, in most of the aforementioned works, the per capita daily adult mortality terms have been restricted to be uniformly positive in order to use the coincidence degree method [3,18,21], fixed point theorems [7,12,13] or comparison principles [9–11,17,19,20,22]. Furthermore, it is difficult to study the global asymptotic behaviour of the Nicholson's blowflies model with variable coefficients and time-varying delays. So far, there has been no result in the literature considering the global existence and global exponential convergence to the zero equilibrium point of positive solutions of nonautonomous Nicholson's blowflies model without the assumption on the uniform positiveness of the per capita daily mortality term.

Motivated by the above discussions, in this paper, we first consider the problem of global existence of positive solutions for a non-autonomous Nicholson's blowflies model of the following form:

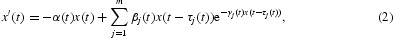

where *m* is a given positive integer, 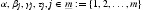, are continuous functions on ℝ^+^, 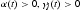 and 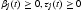 for all 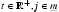. We then employ a novel proof to establish conditions for the global exponential convergence to the zero equilibrium point of model (2). It is worth noting that, the restriction on the uniform positiveness of α(*t*) (that means, there is a positive constant α^−^ such that 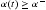 for all *t*≥0) as well as the upper and the lower bounds of 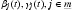, will be removed.

We assume that 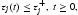 and let 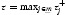. Throughout this paper, let 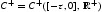 be the set of nonnegative continuous functions with the usual *supremum* norm |.| and 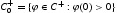. In the biological interpretation of model (2), only positive solutions are meaningful and admissible. Thus we consider only the admissible initial conditions for Equation (2) as follows:

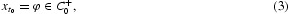

where 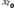 is defined as 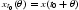 for all 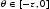.

Note that, the function 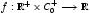 defined as

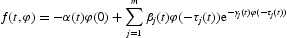

is continuous and locally Lipschitz with respect to 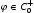. Thus, for each 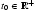, 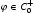, there exists a unique locally solution 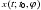 of Equations (2) and (3) (for more details, see [6]). Let 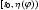 be the right maximal interval of existence of 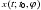.

## 2. Main results

### 2.1. Global existence of positive solutions

In this section we will prove the global existence of positive solutions of Equation (2) for admissible initial conditions (3).

Theorem 2.1 For any 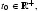 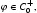 the solution 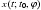 of Equation (2) satisfies

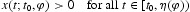

and 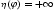.

*Proof* Let 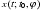 be a solution of Equations (2) and (3). For convenience, let us denote 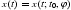 if it does not make any confusion. We will show that

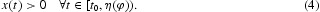

Suppose in contrary that Equation (4) does not hold. Then, there exists 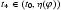 such that *x*(t_*_)=0 and *x*(*t*)>0 for all *t*∈[*t*_0_, t_*_). Thus, *x*(*t*)≥0 for all 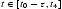. Observing that 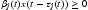 for all 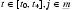, from Equation (2), we have

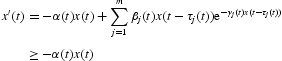

which yields

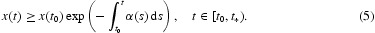

Let 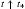, it follows from Equation (5) that, 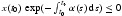, which contradicts with 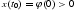. This shows that Equation (4) holds. Consequently, 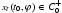 for all 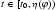. Next, we will prove the global existence of 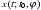, that means 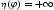. Note that 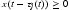 for all 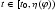. Using the fact 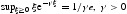, we have

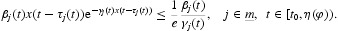

Therefore

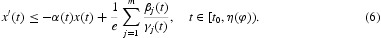

It follows from Equation (6)

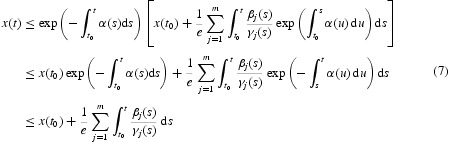

for all 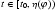. Suppose in contrary that 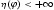, then

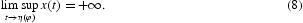

On the other hand, from Equation (7), we have

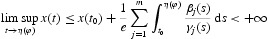

which yields a contradiction with Equation (8). Therefore, 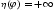. The proof is completed.

*Remark 1* In the proof of Theorem 2.1, we do not require the upper and the lower boundedness of 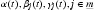, by positive constants as considered in many other results (see, e.g., [3,4,7,9–13,17–22]). Furthermore, if 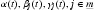 are assumed to be upper and lower bounded by positive constants then the boundedness of any solution 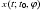 on 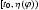 can be proved easily as follows.

For a given bounded continuous function *g*(*t*) on ℝ^+^, let us denote

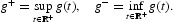

From Equation (7), we obtain

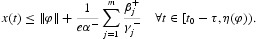

This shows that 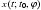 is bounded on 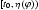 and thus 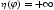 as concluded in Theorem 2.1. As shown in the following example, if α(*t*) is not uniformly positive then positive solutions of Equation (2) may not be bounded.

ExampleConsider the following equation:

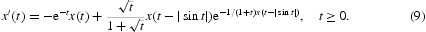

**Fig. 1.**
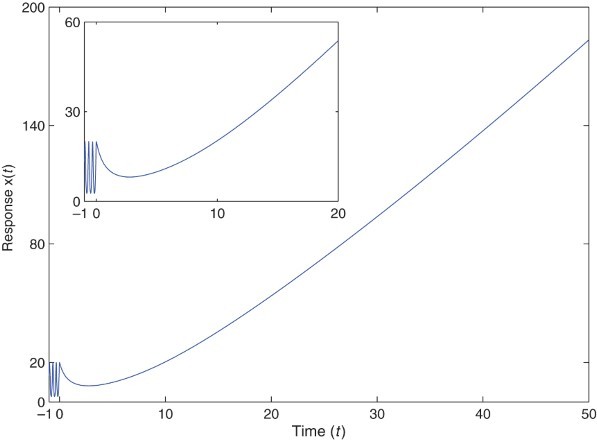
Unbounded state trajectory of (9) with 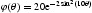.

It can be seen that 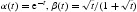 and 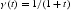 are continuous functions, 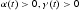 and 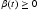 for all 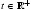 but α(*t*) and γ(*t*) are not uniformly positive. For any 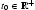, 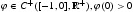, by Theorem 2.1, Equation (9) has a unique positive solution 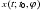 on [*t*_0_,+∞). For illustrative purpose, in the following numerical simulation, we take 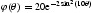 for 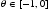. To visualize the effect of initial condition, we simulate the state trajectory from *t*=−1. It is shown in Figure 1 that the corresponding solution 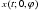 of Equation (9) is unbounded.

### 2.2. Global exponential convergence to the zero equilibrium point

In this section, we will establish conditions for the global exponential convergence to the zero equilibrium point of positive solutions of model (2).

Let us consider the following assumptions:

(A1) There exists *m*[α]>0 such that 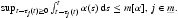

(A2) 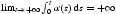.
(A3) 
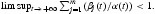

*Remark 2* If α(*t*) is assumed to be upper- and lower-bounded by positive constants then assumptions (A1) and (A2) are obviously removed.

Proposition 2.3 Let assumption (A3) holds. Then there exists a positive constant δ such that any solution 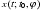 of Equation (2) satisfies

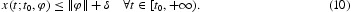

*Proof* By (A3), there exists *T*>0 such that

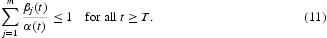

Let us define

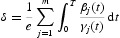

then δ is a positive constant. Suppose 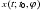 be a solution of Equation (2). Without loss of generality, we assume that *t*_0_≤*T*. From Equation (7), we have

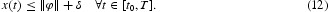

We will prove that Equation (12) holds for all 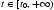. For given ε>0, assume that there exists 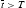 such that

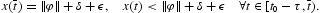

Then, for 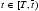, from Equation (2) we have

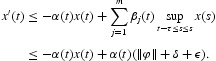

Therefore,

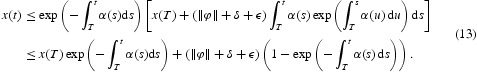

Let 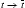, from Equation (13) we obtain

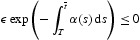

which yields a contradiction. Thus,

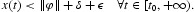

Let 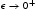 we finally obtain

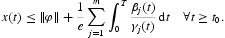

This completes the proof.

*Remark 3* It can be seen from the proof of Proposition 2.3 that, if Equation (11) holds for all 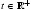 then every solution 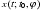 of Equation (2) satisfies 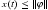 for all *t*≥*t*_0_.

We now prove the global exponential convergence to the zero equilibrium point of positive solutions of Equation (2) as given in the following theorem.

Theorem 2.4 Under assumptions (A1)–(A3), all positive solutions of Equation (2) converge exponentially to the zero equilibrium point of Equation (2). More precisely, there exist positive constants 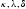 such that every solution 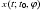 of Equations (2) and (3), with 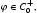 satisfies

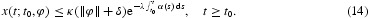

*Remark 4* It is worth noting that, estimation of (14) and (A2) guarantee the global exponential convergence to zero of all positive solutions of (2) which we will refer to *generalized exponential convergence*.

*Proof* Let 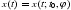 be a solution of Equations (2) and (3). By Proposition 2.3, there exists a constant δ>0 such that

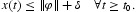

Also, by (A3),

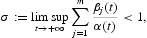

and hence, 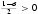. Therefore, there exists T_*_≥*T* (defined in Equation (11)) such that

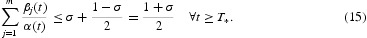

Furthermore, we can assume that 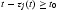 for all 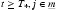. Then, by (A1), we have

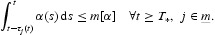



Now, we consider the following scalar equation:

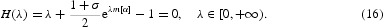

Note that, *H*(λ) is a continuous function, 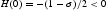, 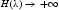 as λ tends to infinity and its derivative 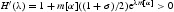 for all 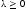. Therefore, Equation (16) has a unique positive solution λ_*_. Moreover, 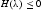 for all 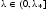. Then, it can be verified that

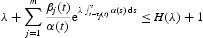

for all *t*≥T_*_, 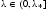, from which we obtain

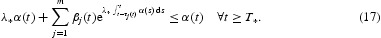

Let us consider the following function:

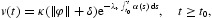

where 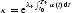. Note that

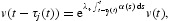

and thus, by Equation (17),

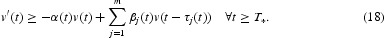

We will show that

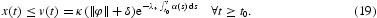

For given ε>0, note that 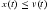 for all *t*∈[*t*_0_, T_*_], we have 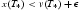. Assume that there exists a *t˜*>T_*_ satisfying 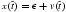, 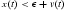 for all 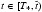. Then, 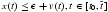. From Equations (2) and (18), we have

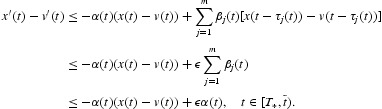

Taking integral on both sides of the above inequality we obtain

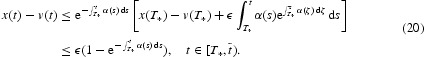

Let 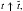 we obtain 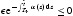, which yields a contradiction. This shows that 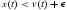 for all *t*≥*t*_0_. Consequently, Equation (19) holds for all *t*≥*t*_0_, and thus, Equation (14) holds for any 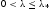. The proof is now completed.

As a special case, if α(*t*) is upper- and lower-bounded by positive constants as considered in many other works in the literature (e.g., [3,11,12,17,20,22]) then we obtain the following corollary.

Corollary 2.5 Assume that (A3) holds and there exist constants 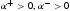 such that 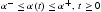. Then every solution 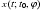 of Equations (2) and (3) satisfies

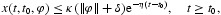

where 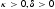 are defined as in the proof of Theorem 2.1 and η is the unique positive solution of the following scalar equation:

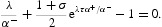

### 2.3. An example

In this section, we give a numerical example to illustrate the effectiveness of our results.

Example 2.6 Consider the following Nicholson's blowflies model with time-varying delay

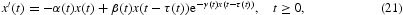

where

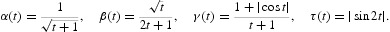

It should be noted that, for this model, the obtained results in the literature cannot be applied to conclude the convergence of positive solutions of Equation (21). In this case we have

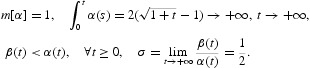

**Fig. 2.**
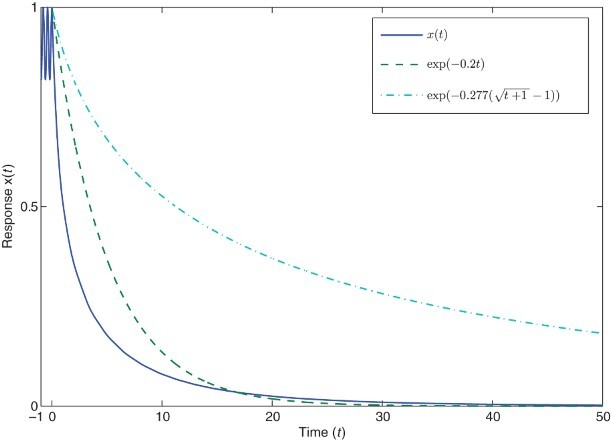
A state trajectory of Equation (21) with 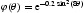.

Therefore, assumptions (A1)–(A3) hold. The scalar equation 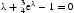 has a unique positive solution 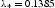. By Remark 3 and Theorem 2.4, every solution 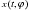 of Equation (21) with 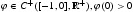, satisfies

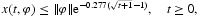

where 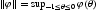. Furthermore, it can be seen that, there is no positive constant η such that 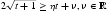, for all *t*≥0. And thus, a classical exponential estimation, that is, 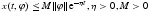, does not exist. Therefore, the exponential estimation proposed in this paper is less conservative and is expected to relax conditions for the exponential convergence of the model. In the following simulation, we take initial function 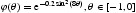. As shown in Figure 2, the corresponding state trajectory of Equation (21) satisfies a generalized exponential estimation 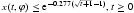. Furthermore, a classical exponential estimation does not exist. For illustrative purpose, we take η=0.2 then it can be seen in Figure 2 that 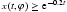 for all *t*≥20.

## 3. Conclusion

This paper has dealt with the global existence and global asymptotic behaviour of positive solutions to a non-autonomous Nicholson's blowflies model with time-varying delays. By using a new approach, we have derived sufficient conditions for the global generalized exponential convergence of positive solutions of the model without any restriction on the uniform positiveness of the per capita dead rate term. Numerical examples have been provided to illustrate the effectiveness of the obtained results.

## Funding

This work was supported by the Ministry of Education and Training of Vietnam (B2013.17.42).
